# Aging promotes reactivation of the Barr body at distal chromosome regions

**DOI:** 10.1038/s43587-025-00856-8

**Published:** 2025-05-01

**Authors:** Sarah Hoelzl, Tim P. Hasenbein, Stefan Engelhardt, Daniel Andergassen

**Affiliations:** 1 Institute of Pharmacology and Toxicology, Technical University Munich, Munich, Germany; 2 DZHK (German Centre for Cardiovascular Research), Partner Site Munich Heart Alliance, Munich, Germany

**Keywords:** Epigenomics, Dosage compensation, Gene regulation, Ageing

## Abstract

Decades ago, evidence of age-related reactivation of a single gene on the female inactive X chromosome was observed in mice. While stable silencing of the Barr body is crucial for balancing gene dosage between sexes, it remains unclear whether silencing is maintained during aging. Here we used allele-specific multi-omics approaches to capture a comprehensive catalog of genes escaping X chromosome inactivation throughout mouse development and aging. We found substantially elevated escape rates during aging across organs, occurring in multiple distinct cell types and concentrated at distal chromosome regions. Consistently, chromatin accessibility was increased across multiple megabases at chromosome ends, affecting regulatory elements of escapees. As several age-specific escapees are linked to human diseases, their elevated expression in females might contribute to sex-biased disease progression observed during aging.

## Abstract (web summary)

Hoelzl et al. employ multi-omics to catalog genes escaping X chromosome inactivation across major organs throughout the mouse lifespan, revealing that aging promotes reactivation of the Barr body at distal chromosome regions.

## Main

In placental mammals, one of the two female X chromosomes is randomly inactivated to compensate for the gene dosage difference between males and females^1^. This process is initiated by the long noncoding RNA *XIST*, which is expressed early in development and coats the entire X chromosome^2^^,3^. *XIST* coating is followed by the recruitment of repressive epigenetic modifiers, such as the Polycomb repressive complexes, and results in an inactive compact chromatin structure known as the Barr body^4,5^. The inactive X chromosome (Xi) is subsequently passed on clonally through mitosis and epigenetically maintained in a silent state^6^. However, a few genes can overcome X chromosome inactivation (XCI), and these so-called escape genes are consequently expressed from both X chromosomes, resulting in higher expression levels in female than in males^7–10^. Thus, escapees are interesting candidates in the context of sex differences observed in physiological processes as well as in various diseases^11–13^.

Mapping escapees in human is challenging due to the random nature of XCI, genetic variation among individuals and limited number of expressed polymorphisms required to differentiate between the two female X chromosomes^14^. By contrast, hybrid mice with fully skewed XCI offer a robust model to detect escape genes from bulk RNA-sequencing (RNA-seq). Furthermore, mouse models serve as an ideal choice for longitudinal studies of escape dynamics, because mice with the same genetic background can be followed over time. So far, only a limited number of adult organs, including brain, spleen and ovaries, have been investigated, showing a range of escape from 3% to 7% (ref. ^15^). These investigations revealed a certain level of organ-specific escape, suggesting variations in the stability of the Xi due to epigenetic differences^15,16^. As epigenetic changes are one of the hallmarks of aging^17^, they may affect the maintenance of the silent Xi during aging. Initial evidence of age-specific reactivation of the Xi in female mice emerged decades ago, but was limited to demonstrating the reactivation of a single gene (*Otc*) in the liver measured using histochemistry^18^.

So far, there has been no systematic investigation of Xi stability throughout the mammalian lifespan. Here, we used allele-specific transcriptomics in a highly polymorphic mouse model with skewed XCI to capture the escape landscape across the major organs throughout development and aging. We found that aging led to a substantial increase in escape rates, concentrated at distal chromosome regions. Age-specific escape correlated with increased chromatin accessibility at regulatory elements of escape genes. These results provide an important basis for future investigations on the contribution of Barr body instability to sex differences in age-related diseases.

## Results

### The escape landscape shows organ and cell type specificity

**Fig. 1:**
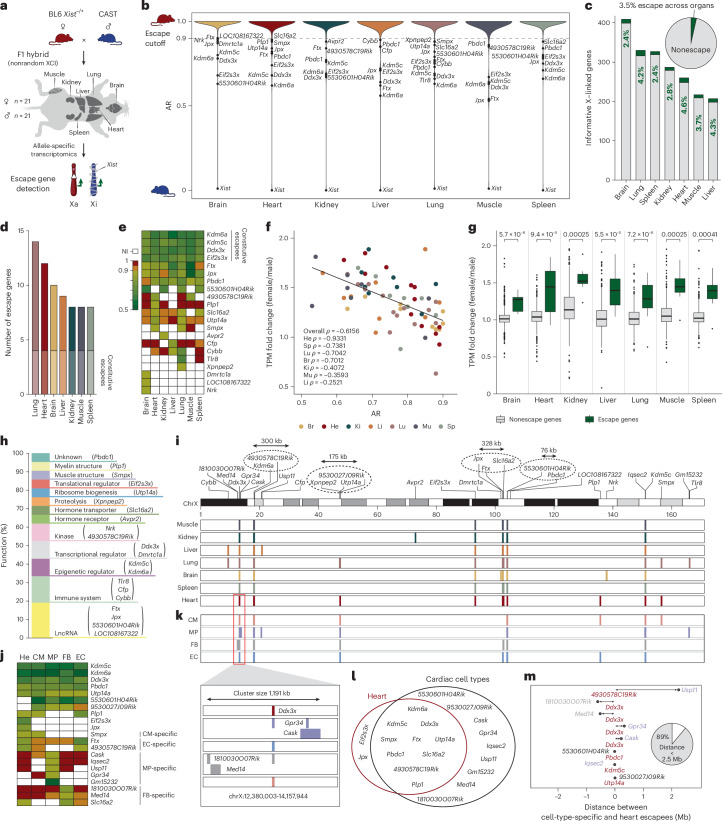
The escape landscape shows a high degree of organ and cell type specificity, with evidence of cluster organization. **a**, A schematic overview of the allele-specific escape detection workflow. Breeding of a BL6 *Xist*^−/+^ mother (red) with a CAST father (blue) results in fully skewed XCI in F1 hybrids. Allele-specific RNA-seq analysis was performed for brain, lung, spleen, liver, heart, kidney and muscle of 9-week-old F1 hybrids (*n* = 3 per sex). **b**, Violin plots showing the median AR of X-linked genes in brain (yellow), heart (red), kidney (turquoise), liver (orange), lung (pink), muscle (purple) and spleen (pastel green). An AR of 1 indicates exclusive maternal expression, while an AR of 0 indicates exclusive expression from the inactive paternal X chromosome. The dotted line represents the cutoff value of ≤0.9 for escape detection. **c**, A bar plot showing all investigated organs ranked by their number of expressed X-linked genes. The percentage of escape is indicated in green. The pie chart shows the mean percentage of escape across all investigated organs. **d**, The number of detected escape genes across all investigated organs. **e**, A heat map showing the median AR of detected escape genes across organs. Red to orange (AR 1 to 0.9) indicates no escape, shades of green (≤0.9) represents escape, and white denotes noninformative genes. **f**, The correlation of median TPM fold change between sexes and median AR of escape genes in the respective organ (colors as assigned above). Normal distribution of the data was assessed using the Shapiro–Wilk test, and the correlation coefficient *ρ* was subsequently calculated using the Spearman method. **g**, A box plot showing the median TPM fold change between sexes of escape genes (green) and nonescape genes (gray) across examined organs. Boxes show the interquartile range (IQR) around the median, and whiskers extend up to 1.5× IQR. Normal distribution of the data was assessed using the Shapiro–Wilk test, and statistical significance between nonescape and escape within each organ was assessed by Mann–Whitney *U* test (two-sided). **h**, Functional roles of detected escape genes (Supplementary Table 2). **i**, Chromosome map depicting the localization of escape genes detected in investigated organs across the X chromosome. Encircled genes show putative cluster formation. **j**, A heat map visualizing median AR of detected escape genes across major cardiac cell types, including cardiomyocytes (CM), macrophages (MP), fibroblasts (FB) and endothelial cells (EC). The color code matches the AR gradient described in **e**. **k**, Localization of escapees detected in the whole heart (red) and major cardiac cell types, including CM (peach), MP (purple), FB (gray) and EC (blue). In the zoom-in window, the putative *Ddx3x* escape cluster is shown. **l**, Overlap of detected escape genes between the whole heart and cardiac cell types. **m**, Distances (Mb) between whole heart escape genes (located at 0 labeled in red) and cardiac cell-type-specific escapees (labeled in the corresponding cardiac cell type color). The pie chart quantifies the proportion of escape genes within and outside 2.5 Mb.

**Extended Data Fig. 1:**
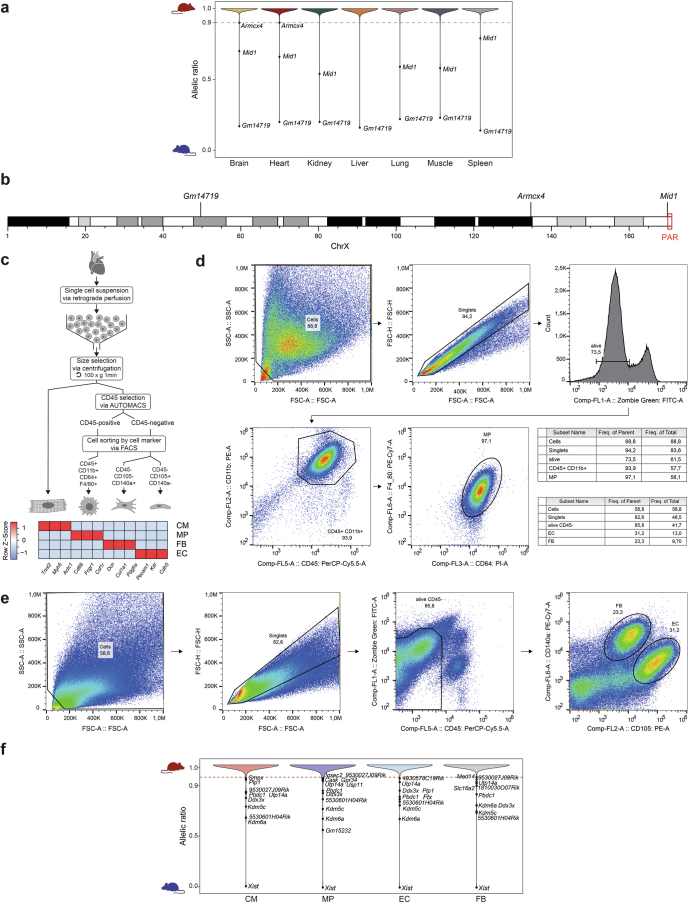
Identification of false positive escape genes and escape characterization of major cardiac cell types. (**a**) Violin plots showing the median allelic ratio of X-linked genes in male organs, including brain (yellow), heart (red), kidney (turquoise), liver (orange), lung (pink), muscle (purple), and spleen (pastel green). Detected escape genes in male tissue were categorized as false positive escape genes and deducted from the female escape analysis. (**b**) Chromosome map depicting the localization of false positive escape genes as well as genes within the pseudoautosomal region (PAR) detected in male. (**c**) Overview of major cardiac cell type isolation workflow. A retrograde perfusion created a single cell suspension of the heart. Cardiomyocytes (CM) were separated by centrifugation at 100 x g for 1 min, creating a CM pellet while non-CM cells remain in the supernatant. Macrophages (MP) were separated from fibroblasts (FB) and endothelial cells (EC) by CD45 magnetic selection via AUTOMACS. Cell marker proteins and cell viability were stained for subsequent cell sorting by FACS (*n* = 3 per cell type). A heat map depicting row Z-scores of marker genes for the corresponding cell types. (**d**) Representative example for the sorting strategy of MP. Viable (Zombie-negative), CD45 + CD11b + CD64 + F4/80+ singlets were sorted. (**e**) Representative example for the sorting strategy of EC and FB. Viable (Zombie-negative), CD45- CD105+ singlets were sorted as EC, while viable (Zombie-negative), CD45- CD140a+ singlets were sorted as FB. (**f**) Violin plots showing the median allelic ratio of X-linked genes in female major cardiac cell types.

To capture a comprehensive catalog of escape genes, we crossed females of the heterozygous *Xist* mouse model^19^ (*Xist*^−/+^, C57BL/6J (BL6) background) with males of the CAST/EiJ (CAST) mouse strain, isolated adult organs from highly polymorphic F1 hybrids (BL6^∆*Xist*^ × CAST), that is, brain, heart, lung, liver, kidney, spleen and muscle, and performed RNA-seq (Fig. 1a). Due to a repA deletion in the long noncoding RNA *Xist* gene on the BL6 X chromosome inherited by the mother, F1 hybrids have fully skewed XCI (BL6 X 100% active). Using an extensive allele-specific expression analysis^20,21^ (Methods), we recovered allele-specific information for an average of 295 ± 71 s.d. X-linked genes across organs (≥20 single-nucleotide polymorphism (SNP)-overlapping reads, 67% ± 5.4% s.d. out of an average of 430 ± 74.1 s.d. expressed X-linked genes with transcripts per million (TPM) ≥1; Supplementary Table 1, sheets a and b). The allelic ratios (ARs) of X-linked genes in our *Xist* mouse model validated the anticipated fully skewed XCI, as most X-linked genes display an AR of approximately 1, confirming that only the maternal BL6 X chromosome is active (Fig. 1b). Genes exhibiting an AR of ≤0.9 were categorized as escape genes, because a minimum of 10% of their expression originates from the paternal Xi. This cutoff was previously used to detect escape genes in mouse and human^7,10^. We excluded genes that showed an AR of ≤0.9 in male samples, as these are probably false positives (*Armcx4* and *Gm14719*) or located within the pseudoautosomal region (*Mid1*) (Extended Data Fig. 1a,b). Across all investigated organs, the percentage of escape ranges from 2.4% in spleen and brain (*n* = 8 and 10 escapees, respectively) to 4.6% in heart (*n* = 12), resulting in a mean of 3.5% escape (Fig. 1c,d). This finding is in line with previous studies that investigated escape patterns in selected mouse tissues^10,15^. Overall, we found a total number of 21 escape genes across all investigated organs, including 9 escapees that were not identified previously (Fig. 1e and Supplementary Table 2)^15^. While some escape genes are shared across all examined organs, including the known constitutive escape genes *Kdm6a*, *Ddx3x*, *Kdm5c* and *Eif2s3x*, 38% of genes escape specifically in one organ (Fig. 1e). Interestingly, eight genes are expressed in multiple organs but escape only in a few selected tissues, suggesting that tissue-specific changes in the epigenetic landscape might have an impact on gene escape. For example, *Plp1* is highly expressed in six out of seven organs, but shows escape only in the heart (Fig. 1e). To assess whether gene escape results in higher expression in females than males, we correlated the AR with the expression fold change between the sexes and observed an overall strong negative correlation (*ρ* = −0.6156; Fig. 1f and Supplementary Table 1, sheet c), suggesting that expression from Xi contributes to a higher gene dose in females. Accordingly, expression fold changes of escape genes are substantially higher compared with nonescape genes across all investigated organs, showing that females have a higher dose of escape genes compared with males (Fig. 1g). This could contribute to sex differences in physiological processes, as escape genes play various roles, including in the musculature (*Smpx*), in immunity (*Tlr8*), in regulating myelin structure (*Plp1*), in epigenetics (*Kdm5c* and *Kdm6a)*, in transcription (*Ddx3x*) and in translation (*Eif2s3x*; Fig. 1h)^22,23^. Evaluation of the localization of the detected escape genes revealed that some escape genes occur alone (for example, *Avpr2*, *Tlr8* and *Smpx*), while others are in close proximity, suggesting cluster organization (Fig. 1i). This is observed for *Kdm6a–4930578C19Rik*, *Xpnpep2–Utp14a–9530027J09Rik* and *5530601H04Rik*–*Pbdc1*, as well as genes within the X inactivation center^24^ (*Jpx*–*Ftx*–*Slc16a2*). To assess the extent of cell-type-specific escape, we focused on the heart and sorted the major cardiac cell types, that is, cardiomyocytes, macrophages, fibroblasts and endothelial cells (Extended Data Fig. 1c–e). Our escape detection workflow validated the majority of escape genes identified in the whole heart (10 out of 12) and identified 9 additional escapees that were previously masked (Fig. 1j–l, Extended Data Fig. 1f and Supplementary Table 1, sheets d and e). Looking at escape at cell-type resolution allowed us to trace which cell type the escape gene originated from. While *Smpx* was initially identified in the whole heart, it exhibits escape exclusively in cardiomyocytes (Fig. 1j,k). Consistent with the observation of organ-specific escape, our results show that escape occurs in a cell-type-specific manner, with some switching from nonescape to escape across cell types, highlighting the influence of cell-type-specific epigenetic mechanisms (Fig. 1j). Notably, the identified cell-type-specific escape genes are in close proximity to escapees identified in the whole heart, with 89% occurring within 2.5 Mb of the whole-heart identified escape genes (Fig. 1k,m). For instance, *Med14*, *1810030O07Rik*, *Cask* and *Gpr34* form an escape cluster with *Ddx3x* in fibroblasts and macrophages (Fig. 1k, zoom-in). In summary, we observed a substantial number of organ and cell-type-specific escape genes that can be organized in distinct clusters.

### Escape rate increases significantly during mouse aging across organs

**Fig. 2:**
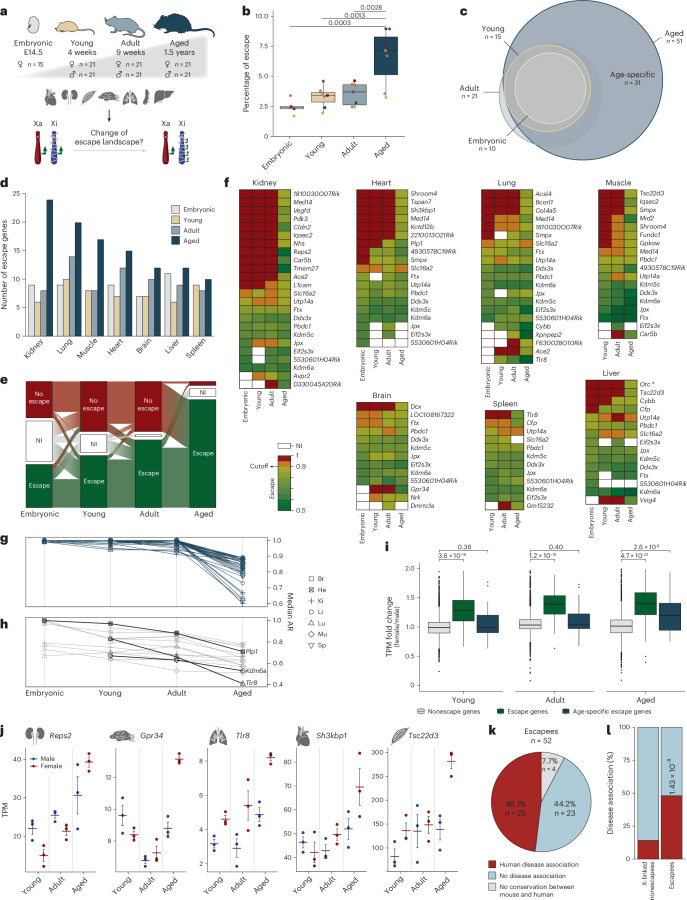
Escape increases during aging across organs. **a**, A schematic overview of detecting the dynamics of escape at four stages: embryonic (gray, E14.5), young (beige, 4 weeks), adult (light blue, 9 weeks) and aged (dark blue, 1.5 years). For the embryonic stage, three replicates were used for female organs, including the heart, lung, brain, kidney and liver. For the young, adult and aged stages, three replicates were used for both male and female organs, including the heart, lung, brain, liver, muscle, kidney and spleen. **b**, The percentage of escape across all investigated organs and time points. Color-coded dots match the colors assigned to organs in Fig. 1. Boxes show the interquartile range (IQR) around the median, and whiskers extend up to 1.5× IQR. Normal distribution of the data was assessed using the Shapiro–Wilk test, and statistical significance was tested using one-way analysis of variance followed by post hoc Tukey’s test. **c**, The overlap of detected escape genes between all four time points. **d**, The number of detected escape genes throughout development and aging across organs. **e**, Dynamics of escape across all four time points. Red, green and white indicate no escape, escape and noninformative (NI), respectively. **f**, Heat maps showing median ARs of detected escape genes across all examined organs at investigated time points. The color code matches the AR gradient described in Fig. 1. The asterisk highlights the first identified age-specific escape gene *Otc* reported by Wareham et al.^18^. **g**, The AR of age-specific escape genes with an ∆AR of ≥0.1 across organs. **h**, Escape genes with a trend toward biallelic expression in aged organs. Genes were selected when they were informative in young, adult and aged and showed an ∆AR of ≥0.1. **i**, The median TPM fold change between sexes of nonescapees (gray), escapees (green) and age-specific escapees (dark blue) across all examined organs at young, adult and aged time points. Boxes show the IQR around the median, and whiskers extend up to 1.5× IQR. Normal distribution of the data was assessed using the Shapiro–Wilk test, and statistical significance was tested using the Kruskal–Wallis test followed by post hoc Dunn’s test with Benjamini–Hochberg correction (two-sided). **j**, TPM expression levels of age-related escape genes between males and females at young, adult and aged time points. Error bars are standard error of the mean. Horizontal lines indicate the mean. **k**, The proportion of escape genes with disease associations (red) and without disease associations (light blue), extracted from the IMPC database (Supplementary Table 1, sheet l). Mouse–human conservation (gray) was extracted from the Mouse Genome Informatics (MGI) and Ensembl databases. **l**, A bar graph illustrating the proportion of disease-associated (red) and non-disease-associated (light blue) escapees and X-linked nonescapees. A Fisher’s exact test revealed a significant enrichment of disease-associated genes among escapees (*P* = 1.43 × 10^−8^, odds ratio 5.60, 95% confidence interval 3.04–10.27, two-sided).

**Extended Data Fig. 2:**
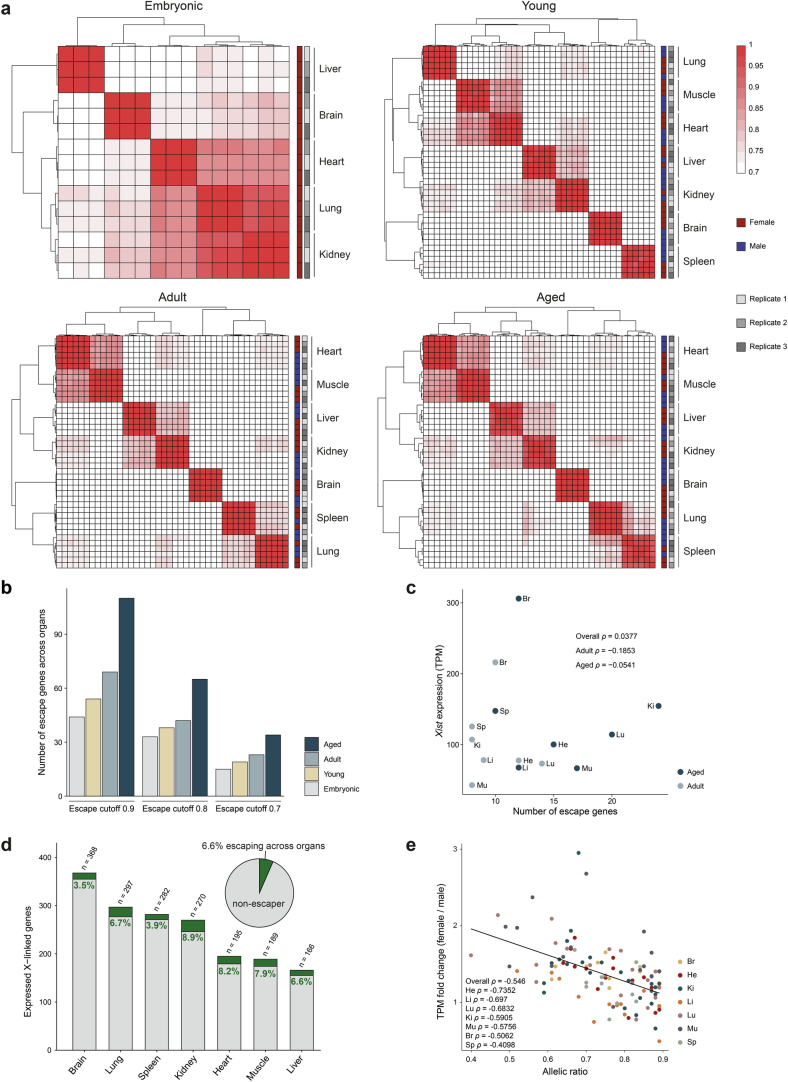
Transcriptome and escape analysis throughout development and aging. (**a**) Unsupervised clustering of a Spearman correlation expression matrix (log10(TPM+1)) across 15 embryonic, 42 young, 42 adult, and 42 aged samples. (**b**) The number of escape genes across all investigated organs is shown for embryonic (gray), young (beige), adult (light blue), and aged (dark blue) time points, based on allelic ratio cutoffs of 0.9, 0.8, and 0.7. (**c**) *Xist* expression levels (measured in TPM) are plotted against the number of escape genes for adult (light blue) and aged (dark blue) organs. Each data point represents an individual organ, with Spearman correlation coefficients calculated separately for adult (*ρ* = −0.1853), aged (*ρ* = −0.0541), and combined datasets (*ρ* = 0.0377). (**d**) Bar plot showing all aged organs ranked by their number of expressed X-linked genes. The percentage of escape is indicated in green. Pie chart showing the mean percentage of escape across all investigated organs at the aged time point. (**e**) Correlation of TPM fold change (female / male) and allelic ratio (AR) of escape genes in the respective organs at the aged timepoint. Normal distribution of the data was assessed using the Shapiro-Wilk test, and the correlation coefficient *ρ* was subsequently calculated using the Spearman method.

Next, we explored the dynamic behavior of escapees throughout development and aging. To accomplish this, we analyzed organs from embryonic stage E14.5, 4-week-old, 9-week-old and 1.5-year-old mice (hereafter referred to as embryonic, young, adult and aged, respectively; Fig. 2a). Unsupervised clustering confirmed sample identity, with replicates of the same organ clustering together (Extended Data Fig. 2a). Using our escape detection workflow, we detected a comparable proportion of gene escape in embryonic (2.5%), young (3.3%) and adult organs (3.5%), with most escape genes shared across the three stages (Fig. 2b,c and Supplementary Table 1, sheets f–k). By contrast, escape significantly increased with age across all organs examined, reaching a mean percentage of 6.6% (*n* = 51 escapees), 31 of which were specifically detected in aged mice (Fig. 2b,c). These escape genes are also expressed at earlier stages from the active X chromosome (Xa) and switch to biallelic expression during aging (Fig. 2e–g). Notably, the increase of escape during aging was independent of the chosen escape cutoff and *Xist* expression levels (Extended Data Fig. 2b,c). The kidney exhibited the highest percentage of escape of 8.9%, reaching a threefold increase (*n* adult = 8, *n* aged = 24; Fig. 2d,f and Extended Data Fig. 2d). A lower number of age-specific escape genes was found in the liver (*n* = 3). This did include the *Otc* gene, the age-specific escapee identified decades ago in the aged liver^18^ (Fig. 2f). Interestingly, several escape genes (*n* = 11) that were not classified as age-specific escapees show a gradual decrease in AR toward biallelic expression, including the constitutive escape gene *Kdm6a* in muscle, *Plp1* in heart and *Tlr8* in lung (Fig. 2h,j). This suggests that an increasing fraction of transcripts originates from Xi with age. Next, we investigated whether age-specific escape leads to higher expression in females compared with males. We found a significant upregulation of age-specific escape genes in aged female organs (Fig. 2i,j and Extended Data Fig. 2e). Interestingly, based on analysis of the International Mouse Phenotyping Consortium (IMPC) database^22^, escape genes are significantly enriched for genes in which mutations lead to disease (*P* = 1.43 × 10^−8^; Fig. 2k,l and Supplementary Table 1, sheet l) and are implicated in several diseases^23^ (Supplementary Table 1, sheet m, and Supplementary Table 2). Therefore, the higher expression of escape genes in aged female organs might indicate that they contribute to the sex bias of age-related diseases.

### Age-specific escape manifests within distinct cell types

**Fig. 3:**
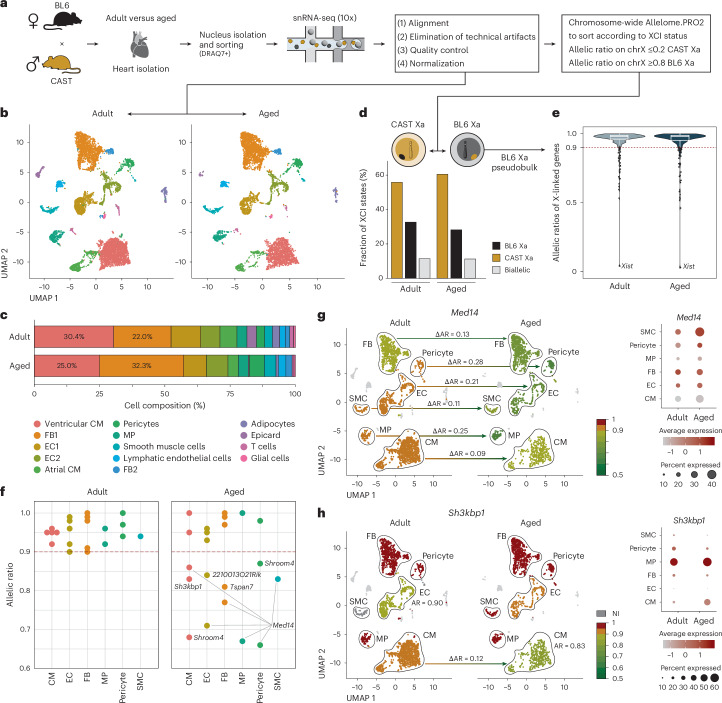
Age-specific escape manifests within distinct cell types rather than arising from population-level shifts. **a**, A schematic overview of the snRNA-seq workflow. Aged and adult hearts were isolated from wild-type BL6 × CAST females (*n* = 2, 1 adult BL6 × CAST, 1 aged BL6 × CAST). Nuclei were isolated and FACS sorted before generating the snRNA-seq libraries using 10x Genomics. After alignment, elimination of technical artifacts, quality control and normalization the whole dataset was analyzed for population shifts. To split the dataset according to the active X chromosome (Xa), a chromosome-wide Allelome.PRO2 analysis was conducted (AR on chrX ≤0.2: CAST Xa; AR on chrX ≥0.8: BL6 Xa). All downstream allele-specific single-nucleus analysis was performed on cells with BL6 Xa only. **b**, UMAP dimensionality reduction of 14 cardiac cell clusters in adult and aged samples. Cell clusters are colored according to **c**. **c**, The cell-type composition in adult and aged samples. **d**, Fractions of cells with BL6 Xa (black), CAST Xa (brown) and biallelic X-linked AR (gray). **e**, A violin plot depicting the AR of X-linked genes of the adult and aged sample obtained by an Allelome.PRO2 analysis using all BL6 Xa cells as a pseudobulk input. Boxes show the interquartile range (IQR) around the median, and whiskers extend up to 1.5× IQR. **f**, The AR of age-specific escape genes identified in the bulk RNA-seq heart. Only cell clusters containing more than 50 BL6 Xa cells in both time points were analyzed (cardiomyocytes (CM), fibroblasts (FB), endothelial cells (EC), pericytes and smooth muscle cells (SMC)). Subclusters of CM, FB and EC were merged for allele-specific analyses. **g**, Left: UMAP visualization showing AR of an example gene *Med14*, across cell clusters during aging. Arrows indicate age-specific escape, with the corresponding delta AR (∆AR) calculated as: AR adult − AR aged. Right: the expression pattern of *Med14* across cell clusters during aging. The color intensity corresponds to the average expression, while the dot size represents the percentage of cells within each cluster expressing *Med14*. **h**, Same as in **g** for age-specific escape gene *Sh3kbp1*.

**Extended Data Fig. 3:**
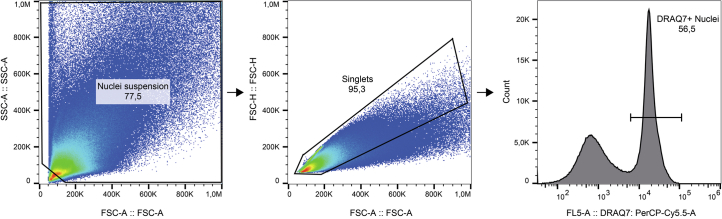
FACS gating strategy to recover cardiac nuclei for snRNA-seq. Representative example for the gating strategy of isolated cardiac nuclei. Nuclei were identified based on size and granularity and DRAQ7+ nuclei were sorted for downstream snRNA-seq.

**Extended Data Fig. 4:**
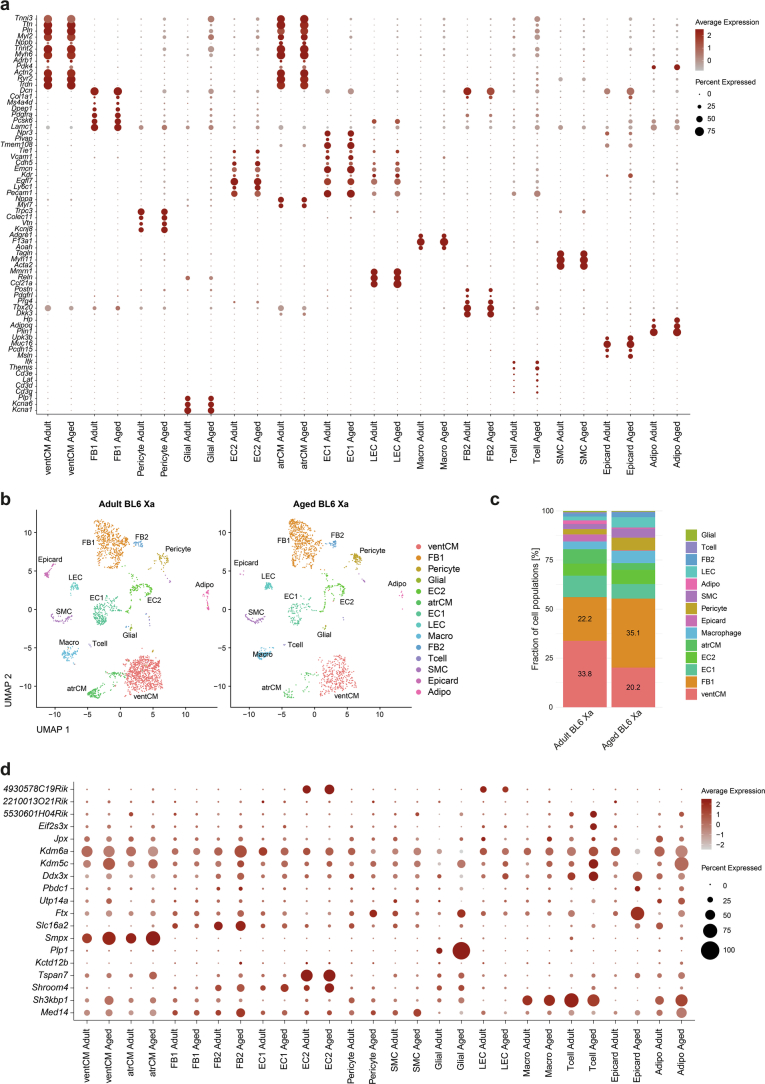
Escape analysis across aging at cardiac cell-type resolution using snRNA-seq. (**a**) Dot plot showing marker gene expression across cell clusters in the heart at the adult and aged time point. The color gradient represents the average expression across the population, while the size of the circle represents the percentage of cells in which this gene is expressed. (**b**) UMAP dimensionality reduction of 14 cardiac cell clusters in adult and aged samples containing only cells with BL6 active X chromosome (Xa). (**c**) Cell-type composition in adult and aged samples containing only cells with BL6 Xa. (**d**) Escape gene expression across cell clusters in the heart at adult and aged time points, containing only cells with BL6 active X chromosome (Xa). The color gradient represents the average expression across the population, while the size of the circle represents the percentage of cells in which this gene is expressed.

**Extended Data Fig. 5:**
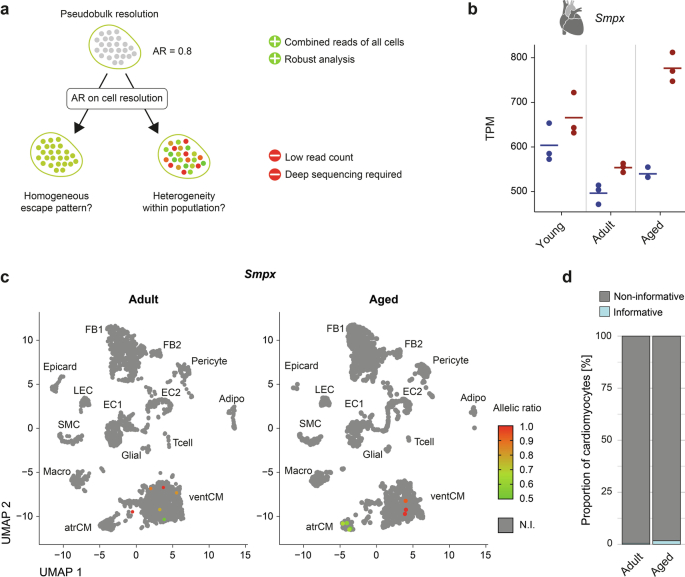
Assessment of escape diversity at cellular resolution for the highest expressed escape gene *Smpx.* (**a**) Schematic overview: The allelic ratio of pseudobulk data could reflect a homogeneous escape pattern across all cells within a population or could represent the average of heterogeneous escape patterns. While pseudobulk analysis combines reads from all cells, providing a robust and reliable overview, allele-specific analysis at the single-cell level faces challenges such as low read counts per cell. Consequently, a deep sequencing approach may be required to obtain reliable results in single-cell allele-specific analyses. (**b**) TPM expression levels of the variable escape gene *Smpx* in males (*n* = 3, blue) and females (*n* = 3, red) in the aging heart. (**c**) UMAP visualization of allele-specific analysis of *Smpx* at single-nucleus resolution. Gray cells represent non-informative cells where the SNP-overlapping total read cutoff ≥ 10 was not met. The color gradient illustrates the allelic ratios of *Smpx* for each single nucleus, ranging from red (AR = 1, inactive) to green (AR = 0.5, biallelic). (**d**) Proportion of cardiomyocytes that are informative (blue) and non-informative (gray) in allele-specific analysis at the single-nucleus level.

**Extended Data Fig. 6:**
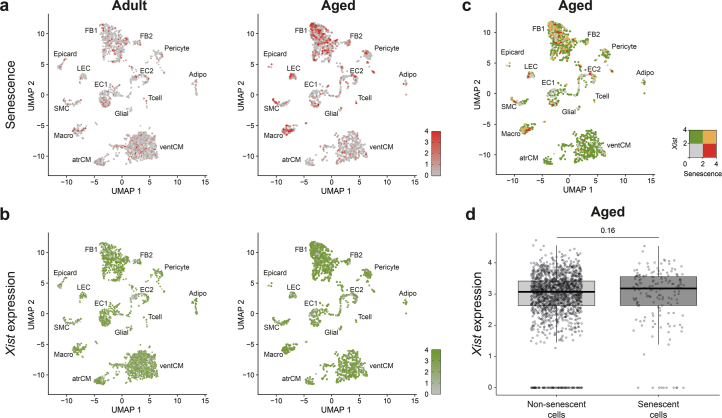
Exploring a role for senescence and *Xist* expression in age-specific escape. (**a**) UMAP visualization showing the z-score of average expression of six commonly used senescence marker genes (*Cdkn1a*, *Cdkn2a*, *Serpine1*, *Trp53*, *Cxcl1*, and *Ccl2*) in adult and aged hearts. (**b**) UMAP visualization displaying expression levels of the lncRNA *Xist*. (**c**) Co-localization plot showing cells positive for both *Xist* and senescence markers (yellow), *Xist* only (green), or senescence markers only (red). Senescent-positive cells were defined as having a z-score of average expression ≥ 2, while *Xist*-positive cells required an expression level ≥ 2. (**d**) Box plot showing *Xist* expression levels of senescent and non-senescent cells. Boxes show the IQR around the median, and whiskers extend up to 1.5× IQR. Normal distribution of the data was assessed using the Shapiro-Wilk test, and statistical significance between non-senescent and senescent cells was assessed by Mann–Whitney *U* test.

The increase in escape during aging observed at the whole-organ level raises the question of whether this phenomenon is due to epigenetic changes that occur in individual cells during aging or due to a shift in cell-type composition. To answer this question, we investigated population shifts and cell-type-specific escape during aging in the heart, an organ with a high number of age-specific escapees. To accomplish this, we isolated F1 hearts (BL6 × CAST) from adult and aged females, extracted the nuclei and sorted them by fluorescence-activated cell sorting (FACS) for subsequent single-nucleus RNA-seq (snRNA-seq; Fig. 3a, Extended Data Fig. 3 and Methods). After quality control and normalization, we obtained 8,127 and 5,986 nuclei for the adult and aged time point, respectively, which were then clustered into 14 cell types (Fig. 3b). Cluster identities were assigned using cell-type-specific marker gene expression (Extended Data Fig. 4a). Examination of cell-type composition during aging showed that the proportion of cardiomyocytes decreased from 30.4% to 25.0%, while the proportion of fibroblasts increased from 22.0% to 32.3% (Fig. 3c and Supplementary Table 1, sheet n). This age-dependent increase in fibroblasts is a well-known phenomenon that ultimately leads to cardiac remodeling by fibrosis in the aged heart^25^. To assess escape in our snRNA-seq data, we first determined the Xa in each individual cell by performing an allele-specific analysis with X-chromosome-wide resolution (Fig. 3a and Methods)^26^. In both adult and aged samples, we observed preferential inactivation of the BL6 X chromosome, a well-known effect in BL6 and CAST hybrids^16,27,28^ (Fig. 3d). For further pseudobulk analysis, we used only cells with an active BL6 X chromosome to ensure comparability with the heterozygous *Xist* model and to avoid the well-documented phenomenon of strain-specific escape (*n* adult = 2,661, *n* aged = 1,730; Extended Data Fig. 4b,c)^16,29,30^. We confirmed that *Xist* was exclusively expressed from Xi (CAST), while the majority of X-linked genes were expressed from Xa (BL6; Fig. 3e). To assign the age-specific cardiac escape genes detected at the whole-organ level to specific cell types, we applied our allele-specific escape detection workflow to cardiac cell clusters using pseudobulk (Supplementary Table 1, sheet o). We also considered investigating escape dynamics and diversity at the cellular level, but our snRNA-seq dataset lacked sufficient SNP-overlapping reads to compute robust ARs per cell, even for the highest-expressed escape gene *Smpx* (Extended Data Fig. 5a–d). Interestingly, we observed genes that show age-specific escape in every cell cluster, but also cell-type-specific escape genes (Fig. 3f). For instance, *Med14* showed an age-related shift across all examined cell populations, with the highest increase of Xi-specific expression observed in pericytes (∆AR = 0.28, Fig. 3g, left). Consistent with this, *Med14* exhibited increased expression levels with age across several cell types (Fig. 3g, right, and Extended Data Fig. 4d). By contrast, *Sh3kbp1* showed a ∆AR of 0.12 specifically in cardiomyocytes, accompanied by an increase in expression (Fig. 3h). We hypothesized that age-specific escape might be driven by changes in *Xist* levels between senescent and nonsenescent cells. However, we found no significant difference in *Xist* expression, suggesting that other mechanisms may be involved (Extended Data Fig. 6a–d). Taken together, our analysis revealed that, despite a shift in cell-type populations in the aging heart, age-specific escape manifests within distinct cell types, indicating that age-related epigenetic changes promote gene escape.

### Age-specific escape is concentrated at distal chromosome regions

**Fig. 4:**
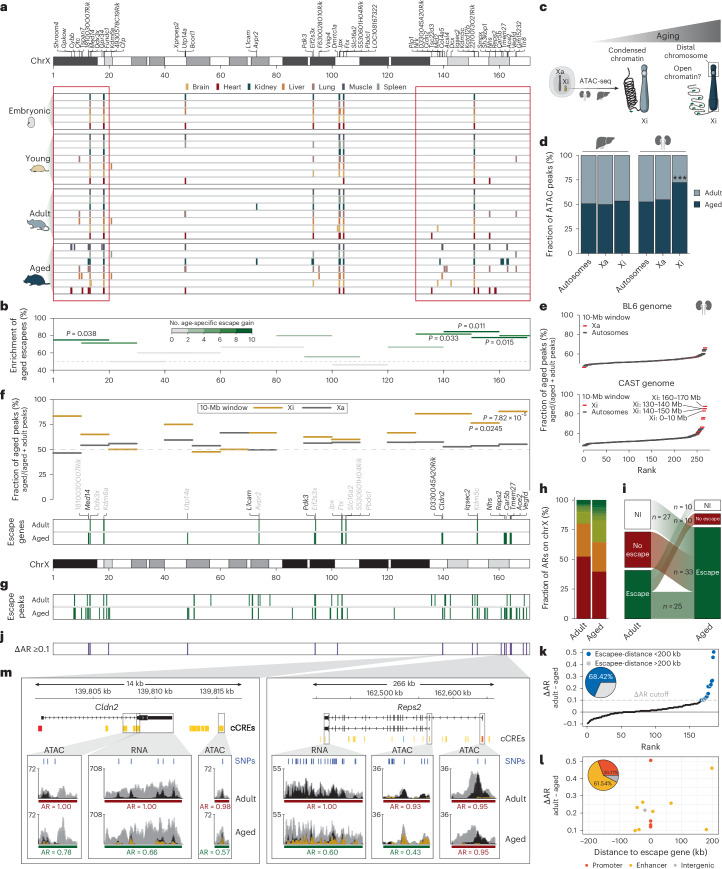
Age-specific escape is enriched at distal chromosome regions with increased chromatin accessibility at regulatory elements of escape genes. **a**, X chromosome map visualizing the localization of escape genes across the X chromosome at all four time points. **b**, Enrichment of escapees identified in aged organs across the X chromosome. Escape genes of all organs were combined, and enrichment was calculated within each 20-Mb sliding window as follows: number of escapees identified in aged organs/total number of escapees in all time points. Bars are colored according to the number of age-specific escape gene gain (gray to green). Significance was determined by a binomial test (two-sided). **c**, An overview of the experimental workflow to investigate whether the X chromosome becomes more accessible with aging using allele-specific chromatin accessibility. Liver (low age-specific escape) and kidney (high age-specific escape) were isolated from F1 BL6^∆*Xist*^ × CAST females (*n* = 2 for both adult and aged samples). **d**, Fraction of ATAC peaks from adult and aged liver and kidney, shown for autosomes, the active (Xa) and inactive (Xi) X chromosomes. Statistical significance was assessed using Fisher’s exact test (two-sided). Asterisks (***) indicate a significant difference with *P* = 1.502 × 10^−6^. **e**, Fraction of aged ATAC peaks in the kidney for each 10-Mb window across the genome, ranked by their enrichment (BL6 allele: upper plot; CAST allele: lower plot). Windows on autosomes are shown in black, while those on the X chromosome are shown in red. **f**, Enrichment of peaks within 10-Mb windows in the aged kidney for Xi (brown) and Xa (gray). Statistical significance was determined by a binomial test (one-sided). Localizations of adult (gray) and age-specific (black) escapees identified in the kidney are shown below. **g**, Localization of escape ATAC peaks with an median AR ≤0.9 in adult and aged kidneys. **h**, Percentages of X-linked median AR in adult and aged samples. The color code matches the gradient described in Fig. 1. **i**, Dynamics of escape ATAC peaks across the adult and aged time point, where red, green and white indicate no escape, escape and noninformative, respectively. **j**, X-chromosome map showing the localization of peaks that are present in all replicates and have ∆AR ≥0.1 (∆AR, AR_adult_ − AR_aged_). **k**, ATAC peaks ranked by ∆AR. Peaks with ∆AR ≥0.1 within 200 kb of an escapee are shown in blue, while those beyond this distance are shown in gray. **l**, The distance between escape genes and ATAC peaks (∆AR ≥0.1 within 200 kb of an escapee), with peak overlap indicated for promoters (red), enhancers (yellow), and intergenic regions (gray). **m**, Genome browser tracks showing the age-specific kidney escape genes *Cldn2* (left) and *Reps2* (right), with Xa-specific reads (black), Xi-specific reads (brown) and non-allele-specific reads (gray) for ATAC- and RNA-seq. Green (escape) and red (nonescape) bars indicate the escape status for genes and ATAC-seq peaks, along with their AR (AR ≤0.9 for escape cutoff). ENCODE candidate *cis*-regulatory elements (cCRES) are shown as: promoter, red; proximal enhancer, orange; distal enhancer, yellow; CTCF, blue. SNPs used for assigning sequencing reads to Xa or Xi are shown in dark blue.

**Extended Data Fig. 7:**
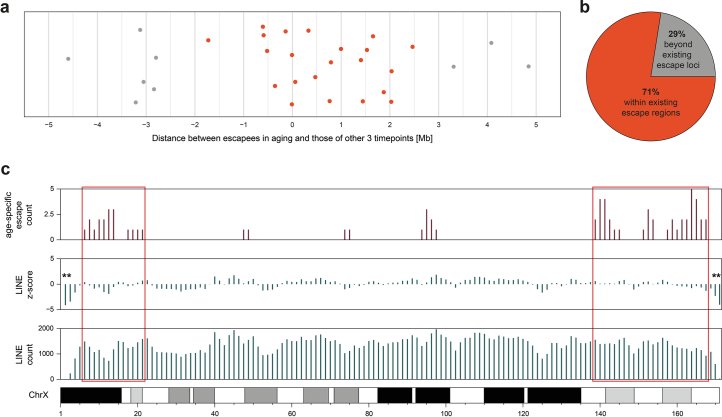
Characterization of age-specific escapees and their clustering patterns across the X chromosome. (**a**) Distances (Mb) between embryonic, young and adult escape genes (located at 0) and age-specific escapees. Dots indicate age-specific escape genes within (red) and beyond (gray) 2.5 Mb of identified escape regions. (**b**) Proportion of age-specific escape genes within (red) and outside 2.5 Mb (gray) of identified escape regions. (**c**) Chromosome-wide visualization of age-specific escape gene counts (top panel), LINE element Z-scores (middle panel), and LINE element counts (bottom panel) calculated using sliding windows of 2.5 Mb. A Z-score cutoff of 1.96, corresponding to a 5% significance level (*p* = 0.05, two-tailed), was applied to determine statistical significance (indicated by asterisks). Red boxes highlight specific regions where most age-specific escape was identified.

Next, we investigated the chromosomal position of age-specific escape genes. This analysis revealed that 71% of these genes are located within 2.5-Mb escape regions identified in earlier stages, while 29% form additional escape loci during aging (Extended Data Fig. 7a,b). This is consistent with our observation that escape genes are organized in clusters. Notably, we did not observe an over- or underrepresentation of long interspersed nuclear elements (LINEs) near escape clusters, DNA elements suggested to promote the spreading of XCI in regions otherwise predisposed to escape (Extended Data Fig. 7c)^31^. Importantly, we found that age-specific escape genes localize predominantly toward the distal ends of the X chromosome (Fig. 4a). Further analysis confirmed that age-specific escapees are significantly enriched at chromosome ends, with the majority (84%) located within the first 20 Mb and the last 40 Mb of the chromosome (Fig. 4b). These findings raise the question of whether the compact and silenced state of the Barr body changes specifically at its distal sites during aging.

### Aging increases chromatin accessibility at distal chromosome regions, affecting regulatory elements of escapees

**Extended Data Fig. 8:**
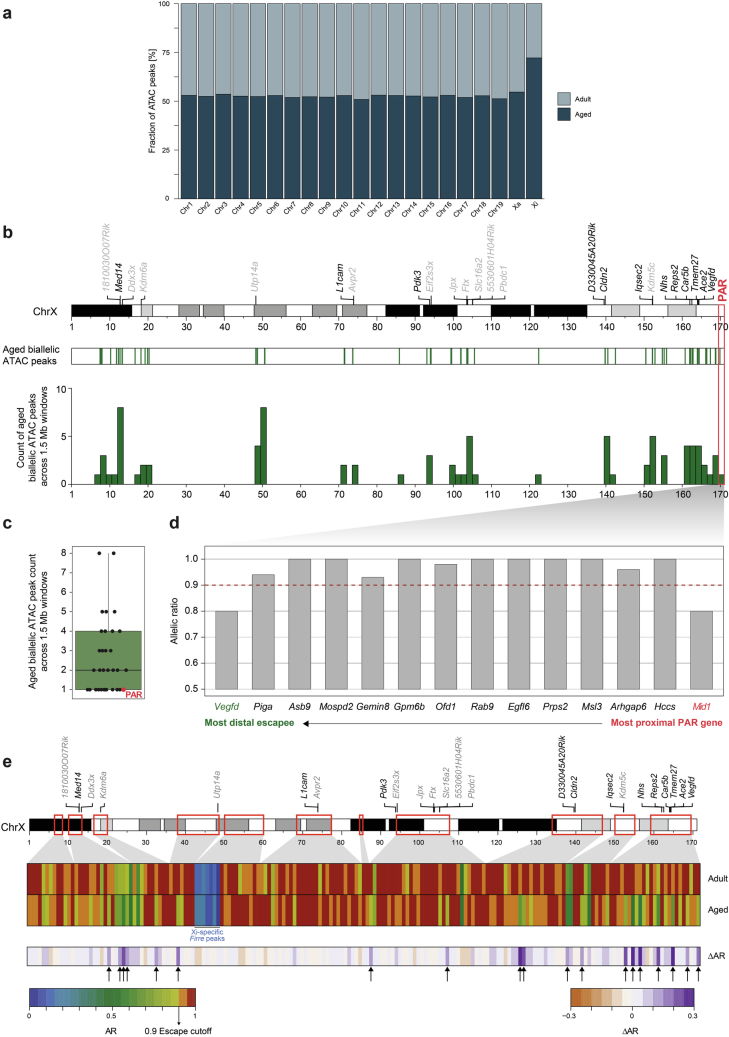
Characterization of chromatin accessibility on the X chromosome in adult and aged kidneys. (**a**) The bar plot illustrates the fraction of ATAC peaks for autosomes (Chr1–Chr19) as well as the active (Xa) and inactive (Xi) X chromosomes in adult (light blue) and aged (dark blue) kidneys. (**b**) Chromosome map showing the pseudoautosomal region (PAR, red) and the localizations of escape genes identified in adult and aged kidneys (top). Escape genes labeled in black are age-specific, while escape genes labeled in gray were detected in adult samples. Distribution of biallelic ATAC peaks along the X chromosome is shown for the aged kidney (middle). The bar plot illustrates the count of aged biallelic ATAC peaks within 1.5 Mb sliding windows, corresponding to the size of the PAR (below). (**c**) Boxplot depicting the distribution of informative aged biallelic ATAC peak counts within 1.5 Mb windows, with the PAR window highlighted. Boxes show the interquartile range (IQR) around the median, and whiskers extend up to 1.5× IQR. (**d**) Allelic ratios of genes adjacent to the PAR in aged kidney samples, spanning from the most distal escape gene (*Vegfd*) to the most proximal PAR gene (*Mid1*). (**e**) A heat map showing median allelic ratios of ATAC peaks in adult and aged kidneys. Escape genes labeled in black are age-specific, while escape genes labeled in gray were detected in adult samples. Delta median allelic ratios (∆AR = AR_adult - AR_aged) were calculated, with the top ∆AR peaks (∆AR ≥ 0.1) indicated by arrows.

**Extended Data Fig. 9:**
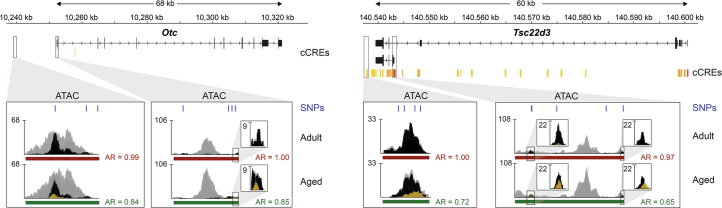
Age-specific escape in the liver is associated with age-specific biallelic ATAC peaks at promoters and nearby enhancers. Genome browser tracks depict allele-specific ATAC-seq data showing age-related accessibility patterns near the identified age-specific escape genes *Otc* (left) and *Tsc22d3* (right). Sequencing peaks for Xa (black), Xi (brown), and non-allele-specific data (gray) are shown. Green and red bars below the genome browser tracks represent escape and nonescape ATAC-seq peaks, respectively (AR ≤ 0.9 as the escape cutoff), along with the computed AR. ENCODE candidate *Cis*-regulatory elements (cCREs) are shown as follows: promoter (red), proximal enhancer (orange), distal enhancer (yellow), and CTCF (blue). SNPs used for assigning sequencing reads to Xa or Xi are shown in dark blue.

To determine whether the condensed Barr body loosens during aging at distal chromosome regions, we assessed chromatin accessibility. Thus, we generated assay for transposase-accessible chromatin using sequencing (ATAC-seq) libraries from adult and aged organs, specifically the liver, which has a low number of age-specific escapees, and the kidney, which has the highest number (Fig. 4c). In both organs, the number of ATAC peaks on the Xa as well as on autosomes did not significantly change with age (Fig. 4d, Extended Data Fig. 8a and Supplementary Table 1, sheet p). By contrast, the fraction of ATAC peaks on the Xi increased significantly with age in the kidney (*P* = 9.238 × 10^−6^), but not in the liver (Fig. 4d). This result indicates that the Xi is more accessible in the organ that features a higher number of age-specific escapees. To determine the regions on the Xi responsible for increased age-specific accessibility in the kidney, we calculated the ratio between adult and aged ATAC peaks over 10-Mb windows and found an overrepresentation at the distal regions of the Xi, overlapping with age-specific kidney escapees (Fig. 4e,f). Consistently, we observed a higher proportion of biallelic ATAC-seq peaks enriched at distal chromosome regions in the aged kidney, a pattern that cannot be explained by the spreading of an open pseudoautosomal region (Fig. 4g,h, Extended Data Fig. 8b–d and Supplementary Table 1, sheet q). These biallelic peaks consist of 27 de novo age-specific peaks and 33 peaks that switch from Xa-specific to biallelic during aging (Fig. 4i). To determine the most robust shifts during aging, we filtered for peaks shared across replicates that were at least 10% more accessible on Xi in the aged samples. This narrowed it down to 19 high-confidence ATAC-seq peaks, which were mostly concentrated at distal chromosome regions and found in proximity of escape genes (68.42% within 200 kb of escapees; Fig. 4j,k, and Extended Data Fig. 8e). Among these, 92.31% overlap with regulatory elements such as escape gene promoters (30.77%) and nearby enhancers (61.54%; Fig. 4l). A notable example is the age-specific escape gene *Cldn2*. We detected two age-specific accessible regions, which overlap enhancers, one within the gene body of *Cldn2* and another approximately 4 kb downstream, probably controlling expression from the Xi (Fig. 4m, left). Another interesting example is *Reps2*, where a specific isoform escapes in an age-dependent manner. While the long isoform of *Reps2* is expressed from the Xa, the promoter of the short isoform shows age-dependent biallelic activity, leading to Xi-specific expression of the short isoform (Fig. 4m, right). We further investigated the age-related accessibility of the three age-specific escapees identified in the liver and observed biallelic peaks at promoters and enhancers in two genes, suggesting that this phenomenon may occur across organs (Extended Data Fig. 9). Our in-depth, allele-specific characterization shows that, during aging, the distal chromosome regions of the Xi become more accessible at sites of regulatory elements that might drive escape gene expression.

## Discussion

In our systematic analysis of escape during development and aging, we observed that gene escape substantially increases with age in all examined organs. This suggests that maintenance of the silent Barr body is impaired with age. Progressive destabilization of the inactive X chromosome during aging was hypothesized decades ago based on the observation of a single escape gene (*Otc*) in the liver^18^. However, at the time, the authors could not determine whether the reactivation of this gene was an isolated event or indicated a reversal of XCI patterns^18,32^. Our study demonstrates that Xi reactivation during aging is widespread. Specifically, we observed an increase in escape from an average of 3.5% in the adult stage to 6.6% in the aged stage out of informative X-linked genes across organs. Using allele-specific snRNA-seq analysis, we showed that age-specific escape occurs in a cell-type-specific manner, indicating that epigenetic changes during aging might drive gene escape. Accordingly, we observed an age-related increase in chromatin accessibility on the Barr body, overlapping DNA regulatory elements in proximity to escape sites, suggesting that these elements might allow age-specific escape. Given that epigenetic alterations are one of the hallmarks of aging^17^, our results are in line with recent studies that observed DNA methylation changes on the X chromosome during aging in a sex-specific manner^33–36^. Strikingly, we discovered that age-specific escapees and chromatin accessibility are enriched at distal chromosome regions. Previous studies have described a role for telomeres in regulating gene expression over multiple megabases via telomere loops on autosomes^37,38^ and in reactivating a single X-linked gene^39^. Whether telomere shortening plays a role in age-specific reactivation and how aging affects different layers of the epigenome, leading to Barr body instability, require further investigation.

Our study provides a comprehensive escape atlas across multiple organs during the murine lifespan. We extended the observation of organ-specific gene escape^15^ to the major organs and discovered cell-type-specific escape in the heart. We also uncovered that escape is organized in distinct gene clusters, which is consistent with observations in mice^40,41^ and humans^7^. Cluster organization suggests a common regulatory mechanism that controls gene escape in a cell-type-specific manner, but whether specific motifs for DNA binding proteins regulate escape warrants further investigation^14,42^. One limitation of this study is that only 81% of X-linked genes can be assessed due to the presence of SNPs within the gene body. However, from the remaining 19%, the majority was not expressed in our dataset. Nevertheless, we were able to assess the escape status of a substantial fraction of expressed X-linked genes, representing a robust dataset for further analysis.

Whether age-specific escape also occurs in humans remains to be discovered. Conducting a longitudinal studies in humans is highly challenging due to the difficulty of obtaining samples from the same individual over time. Moreover, mapping escape in human is complicated because of the random nature of XCI, genetic variation among individuals and the limited number of expressed polymorphisms^14^. A previous study mapped human escape genes in postmortem samples by comparing expression between sexes, analyzing one fully skewed XCI female and performing allele-specific single-cell analyses, revealing escape of 23% of assessed X-linked genes, with a high degree of tissue specificity^9^. However, a recent study analyzed organs from the same resource and described tissue-specific escape as a rare event^43^. The resulting discrepancy between these studies highlight the challenges of escape determination in humans^43^. Despite this incomplete human escape landscape, a handful of murine escape genes also exhibit escape in humans, implying that the phenomenon of escape is partially conserved^7,10^. Thus, our mouse atlas (Supplementary Table 2) provides the foundation to assess the extent of escape conservation between mice and humans, as gene dosage conservation across species may suggest functional importance (Supplementary Table 1, sheet r).

The highly conserved X chromosome contains over a thousand genes, many of which are linked to human diseases^22,23^. Our escape gene catalog also reveals a significant enrichment for disease-relevant genes. This finding lays the groundwork for future investigations into the causes of sex bias in disease, which is currently attributed mainly to hormonal differences. For example, evidence for a direct link between gene escape and female-biased autoimmunity has been demonstrated for *TLR7* and *TLR8*, with their higher escape gene dose leading to enhanced immune responses^44–46^. In line with these observations, we identified *Tlr8* as an escape gene in organs with a high fraction of immune cells. Interestingly, *Tlr8* exhibited a gain of escape with age, accompanied by higher expression in females, which may contribute to age-related autoimmune disorders such as late-onset lupus^47^. In line with our observation of increased *Plp1* escape during aging in cardiac glial cells, a recent study reported a similar pattern in the aging hippocampus and showed that *Plp1* overexpression improved cognition, highlighting the beneficial role of age-related escape^48^. Another interesting example is *Ace2*, which shows age-specific escape in the lung and has been shown to reduce pulmonary fibrosis^49^. This condition has a higher prevalence in men, with risk increasing with age, indicating that the higher expression of *Ace2* in females may offer protection^49–51^. Future studies will need to address the role of age-related escape genes in sex-specific disease progression during aging and whether they could be implicated in lifespan differences between sexes.

In conclusion, our systematic assessment of Barr body stability identified age-specific reactivation at distal chromosome regions, which probably promotes gene escape. The subsequent increase in expression of conserved and disease-relevant genes in females might provide additional insights into sex-specific disease mechanisms, complementing the role of hormones in explaining sex biases in age-related disease.

## Methods

### Mouse strains

Mice were housed under a 12-h light/dark cycle at 21 ± 1 °C with 40–70% humidity at the Technical University of Munich, Institute of Pharmacology and Toxicology. BL6 *Xist*^−/+^ females were purchased from Riken BRC Japan (RBRC02655: B6;129-Xist<tm5Sado>) and were maintained by breeding with wild-type C57BL/6J purchased from Jackson Laboratory (JAX: strain #000664). BL6 *Xist*^−/+^ females and wild-type CAST males (JAX: strain #000928) were used for breeding to obtain experimental F1 hybrids. Mice were weaned and genotyped at 4 weeks of age (genotyping primers are listed in ref. ^19^). All animal experiments were performed in accordance with relevant guidelines and regulations, including the EU guideline 2010/63, the German Animal Welfare Act and ARRIVE guidelines. Approval was granted by the authorities (District Administration Department of the City of Munich, Veterinary Office Munich City, permit according to §11, paragraph 1 sentence 1 no. 1 of the Animal Welfare Act).

### Organ isolation

At 4 weeks, 9 weeks and 75 ± 4 weeks (1.5 years), F1 hybrids were euthanized via cervical dislocation and organs (brain, heart, liver, lung, kidney, spleen and muscle) were collected and flash-frozen in liquid nitrogen. Organs were stored at −80 °C for further use. Three replicates per time point and sex (BL6^∆*Xist*^ × CAST females and BL6 × CAST males) were analyzed.

For embryonic organ isolation, BL6 *Xist*^−/+^ females and wild-type CAST males were mated, and females were checked for a vaginal plug the next morning. At E14.5, embryos were isolated. Organs (brain, heart, liver, lung and kidney) were collected and flash-frozen in liquid nitrogen. Organs were stored at −80 °C for further processing. Three replicates of BL6^∆*Xist*^ × CAST females were analyzed.

### RNA isolation and library preparation for high-input bulk RNA-sequencing

Frozen organs of 4-week-, 9-week- 1.5-year-old mice were homogenized in appropriate amounts of TRIzol reagent (1 ml TRIzol reagent per 50–100 mg tissue) using the GentleMACS Dissociator RNA_02_01 program. One milliliter of homogenized organs was used for RNA isolation. Frozen organs of E14.5 embryos were thawed in TRIzol reagent and homogenized using a pellet pestle. RNA isolation was performed for all time points according to the manufacturer’s instructions (Invitrogen, TRIzol reagent, cat. no. 15596018). RNA concentration was measured by the NanoDrop Microvolume Spectrophotometer. Then, 100 ng of RNA was used to generate poly-A captured RNA-seq libraries using lllumina’s Stranded mRNA Prep, Ligation kit according to manufacturer’s instructions. Concentration and fragment distribution of completed RNA-seq libraries was measured on Agilent’s TapeStation System. Libraries were sequenced in the 50-bp paired-end mode on a NovaSeq6000 at Helmholtz Munich.

### Major cardiac cell type isolation and library preparation for low-input bulk RNA-sequencing

To identify escape genes on cell-type resolution, hearts of three replicates per sex (BL6^∆*Xist*^ × CAST females and BL6 × CAST males) were isolated. To obtain a single-cell suspension of the heart, an enzymatic retrograde perfusion was performed. To do so, the heart was collected and cannulated at the aorta. Coronary arteries were flushed briefly with 1 ml perfusion buffer (for reagents for buffer compositions, see Supplementary Table 1, sheet s). Afterward, the cannula with the heart was attached to the perfusion pump system, and the heart was perfused with perfusion buffer for 1 min (4 ml min^−1^). Next, the heart was rinsed with a digestion buffer for 10 min (recirculating, 4 ml min^−1^) to enzymatically dissociate the ventricular cells. Then, the heart was detached, atria were discarded and the ventricles were roughly homogenized in 2.5 ml digestion buffer by cutting with scissors. A 1-ml syringe was used to shear the tissue pieces up and down 30 times. After adding 2.5 ml stop buffer, the syringe sheared the liquid again 30 times. Ultimately, the suspension was filtered through a 100-µm strainer and put on ice.

To separate cardiomyocytes from noncardiomyocytes, the suspension was centrifuged at 100*g* at 4 °C for 1 min and supernatant was transferred. This was repeated once and the sediment, containing cardiomyocytes, was resuspended in TRIzol, homogenized using a pellet pestle, and frozen at −80 °C for further use. The supernatant, containing noncardiomyocytes, was spun down at 400*g* at 4 °C for 7 min. After discarding the supernatant, the pellet was resuspended in red blood cell lysis buffer at room temperature for 0.5 min. Next, 2 ml PBS was added, and suspension was centrifuged at the same settings, followed by the filtering through a 70-µm strainer. Cells were blocked with Fc-block at 4 °C for 15 min, before using CD45-conjugated microbeads (Miltenyi) to bind CD45-positive cells (Supplementary Table 1, sheet t). Afterward, the cell suspension was separated into a CD45-positive and CD45-negative fraction using the autoMACS Pro Separator by Miltenyi. Then, both fractions were stained with Zombie Green to detect dead cells, followed by the macrophage staining for the CD45-positive fraction (CD45-positive, CD11b, CD64, F4/80) and the endothelial staining (CD45-negative, CD105) and the fibroblast staining (CD45-negative, CD140a) for the CD45-negative fraction (Supplementary Table 1, sheet t). After washing twice, 2,000 cells of each cell type were sorted, immediately frozen on dry ice and stored at −80 °C for further use. After thawing, sorted major cardiac cell types were resuspended in 1 ml TRIzol and RNA was isolated according to manufacturer’s instructions (Invitrogen, TRIzol reagent, cat. no. 15596018). Because the starting sample was small, 10 μg of RNase-free glycogen was used as a carrier, and isopropanol precipitation was performed at −20 °C overnight. RNA quality was measured on Agilent’s TapeStation system. Then, 0.5–3 ng of RNA were used to generate RNA-seq libraries using Takara’s SMART-Seq v4 PLUS kit according to the manufacturer’s instructions. The concentration and fragment distribution of completed RNA-seq libraries was measured on Agilent’s TapeStation system. Libraries were sequenced in the 50-bp paired-end mode on a NovaSeq6000 at Helmholtz Munich.

### Nuclei isolation and library preparation for ATAC- and snRNA-seq

Nuclei were chosen as an input. On the one hand, this allowed us to include cardiomyocytes in our snRNA-seq analysis that as viable cells do not fit into the 10x Genomics microfluidic channels. On the other hand, it reduced the mitochondrial read fraction in ATAC-seq experiments. To isolate nuclei from frozen tissue, the nuclei extraction buffer (Miltenyi) was used according to manufacturer’s instructions. For ATAC-seq, the integrity of isolated nuclei was assessed on the Countess II FL Automated Cell Counter equipped with the DAPI EVOS light cube. In total, 50,000 nuclei of 2 replicates (BL6^∆*Xist*^ × CAST females) per time point were taken as input for Active Motif’s ATAC-Seq kit, and libraries were generated according to manufacturer’s instructions. ATAC libraries were sequenced on a NovaSeq6000 at Helmholtz Munich.

For snRNA-seq, nuclei of 1 replicate per time point (BL6 × CAST females) were incubated with DRAQ7 for 5 min before sorting, staining the DNA of nuclei. Sorting the DRAQ7-positive population ensured a debris-free nuclei suspension, which was again assessed on the Countess II FL Automated Cell Counter equipped with the DAPI EVOS light cube. Single-nucleus libraries were generated using the Chromium Next GEM Single Cell 3′ Reagent Kit v3.1 (10x Genomics) with a target of 10,000 nuclei per sample. The single-nucleus libraries were prepared according to the manufacturer’s instructions. Sequencing was performed on a NovaSeq6000 at Helmholtz Munich.

### RNA-seq analysis

After quality assessment using the FASTQC software tool (version 0.11.9)^52^, RNA-seq data were mapped to the mouse reference genome GRCm38/mm10 (ref. ^53^) by the STAR aligner (version 2.6.0c)^54^. For downstream analysis, multimappers and alignments containing noncanonical junctions as well as intron sizes >100,000, were excluded. Quantification of uniquely aligned reads was performed by htseq-count (version 0.11.3)^55^. A custom Perl script was used to further separate strand-specific information. SNPsplit (version 0.6.0)^56^ was performed to separate reads to their respective alleles for visualization of allele-specific RNA expression in genome browser tracks. TPM were computed by a custom R script to compare male and female expression levels.

### ATAC-seq analysis

Alignment of ATAC-seq data was performed using the bowtie2 aligner in paired-end mode with the parameter ‘--very-sensitive’^57^. To obtain high-quality reads for downstream analysis, mitochondrial reads, mapping artifacts (≤38 bp or ≥2,000 bp), low-quality reads (mapping quality (MAPQ) <20), ENCODE blacklist genes (blacklist.v2.bed)^58^ and duplicates (identified by GATK MarkDuplicates, version 4.2.2.0)^59^ were removed. This left at least 33 million high-quality mapped reads for downstream analysis. To investigate differences in the adult and aged chromatin profile of Xa and Xi, SNPsplit assigned reads to their corresponding allele^56^. MACS2 (version 2.1.0) was used to call broad peaks^60^, which were then counted within 10-Mb windows along the entire genome. The median number of peaks per window was calculated across replicates, and the enrichment of aged peaks was calculated by dividing the number of aged peaks by the number of total peaks (aged + adult peaks). To test for significance of peak enrichment during aging, a binomial test was applied, assuming an equal number of peaks between the time points.

### Allele-specific analysis for RNA-seq and ATAC-seq data

To obtain allele-specific information on RNA expression and chromatin accessibility, we used the updated Allelome.PRO2 pipeline^20,21^.

For the allele-specific RNA-seq analysis of whole organs, the GRCm38/mm10 RefSeq gene annotation^53^ was used together with the previously documented SNP dataset^61^, covering 20,635,313 SNPs between CAST and BL6 strains. A minread (minimum number of reads covering a SNP) cutoff value of ≥1 and a total read cutoff value (total read count covering a gene) of ≥20 were applied.

For the allele-specific RNA-seq analysis of major cardiac cell types, the same annotation was used in combination with a SNP dataset, containing only SNPs located in exonic regions. Here, a minread cutoff value of ≥1 and a total read cutoff value of ≥10 were applied.

Our escape detection workflow identified X-linked genes with an AR ≤0.9 in females as escape genes. X-linked genes with an AR ≤0.9 in males were excluded from the analysis, including the following genes from the pseudoautosomal region: *Mid1*, *G530011O06Rik* and *Gm39551*; and four additional false-positive genes outside the pseudoautosomal region: *Gm14719*, *Armcx4*, *Sms* and *Llph-ps2*.

For the allele-specific ATAC-seq analysis, an annotation was created by intersecting the peaks called across time points and replicates^62^. In combination with the complete SNP dataset also used for RNA-seq data from organs, Allelome.PRO2 was conducted with a minread cutoff value of ≥1 and a total read cutoff value of ≥20. In addition, the Allelome.PRO2 output was filtered for only those peaks that were called by MACS2 in their respective sample.

### snRNA-seq data preprocessing

The cellranger tool (Cell Ranger version 7.1.0, 10x Genomics)^63^ was used to align reads, quantify gene expression and call cells of raw snRNA-seq data. Subsequently, CellBender (version 0.3.0) was performed with default settings to eliminate technical artifacts such as ambient RNAs, which are common in single-nucleus data^64^. Subsequent downstream processing of filtered count matrices was done in R using the Seurat package (version 5.1.0)^65^. Quality control further filtered for cells with 500 < nCount < 20,000, 200 < nFeature < 5,000, and a percentage of mitochondrial reads >5. In addition, genes expressed in ≤10 cells were discarded. To remove doublets, the DoubletFinder package (version 2.0.3) was used according to their instructions^66^. Samples were normalized using the SCTransform package (version 0.4.1)^67^, while the percentage of mitochondrial reads was simultaneously regressed out. The Seurat objects were then integrated by Seurat’s IntegrateData() function and principal components were computed by RunPCA(), using the top 40 dimensions for UMAP dimensional reduction and clustering. Based on the expression of marker genes, individual cell clusters were manually annotated.

### Allele-specific snRNA-seq analysis

To determine whether the CAST or the BL6 X chromosome is active in a single cell, we bioinformatically sorted each cell according to the XCI status as previously described in ref. ^26^. In brief, a chromosome-wide Allelome.PRO2 run was conducted for each individual cell. The CellRanger output bam file was separated into single cell bam files using the command filterbarcodes within the sinto package^68^. A custom-made chromosome-wide annotation together with a SNP dataset, which excluded SNPs covering the *Xist* locus, was used to perform Allelome.PRO2. Only cells containing ≥10 X-chromosomal reads were further analyzed. To determine their XCI status, cells were then categorized on the basis of their AR (AR ≤0.2 CAST Xa, AR ≥0.8 BL6 Xa, 0.2 ≤ AR ≤ 0.8 biallelic).

To confirm age-specific escape genes in snRNA-seq cell clusters, an allele-specific pseudobulk analysis was performed, separating the CellRanger output bam file into pseudobulk bam files on the basis of their cluster and Xa status. To compare the snRNA-seq results with our bulk RNA-seq obtained from BL6^∆*Xist*^ × CAST females and to minimize strain-specific escape, only cells with a BL6 Xa were analyzed as a pseudobulk. The subsequent Allelome.PRO2 analysis used the same annotation as well as the SNP file as described for the whole-organ bulk RNA-seq data. Only cell clusters containing more than 50 cells in both time points were analyzed. The minread cutoff was set to 1, while the total read cutoff was ≥20.

### Statistics and reproducibility

Sample sizes were determined on the basis of previous experiments^16,29^. The experiments were not randomized. For in vivo sequencing data like in this study, blinding was not necessary, as the experimenter has no influence on the results of these tests. No data were excluded from the analyses. Normality of the data was tested using the Shapiro–Wilk test. Statistical significance was assessed as indicated in the figure legends.

### Reporting summary

Further information on research design is available in the Nature Portfolio Reporting Summary linked to this article.

## Supplementary information

Reporting Summary

Supplementary Table 1 Multi-omics investigation to map and characterize gene escape during mouse aging. **a**, Sample information from of all sequenced samples. **b**, ARs of X-linked genes across adult organs. **c**, Expression levels (TPM) of X-linked genes across adult organs. **d**, ARs of X-linked genes across cardiac cell types. **e**, Expression levels (TPM) of X-linked genes across cardiac cell types. **f**, ARs of X-linked genes across embryonic organs. **g**, Expression levels (TPM) of X-linked genes across embryonic organs. **h**, ARs of X-linked genes across young organs. **i**, Expression levels (TPM) of X-linked genes across young organs. **j**, ARs of X-linked genes across aged organs. **k**, Expression levels (TPM) of X-linked genes across aged organs. **l**, Disease association of identified escape genes retrieved from IMPC. **m**, Disease implications of identified escape genes retrieved from the Alliance of Genome Resources Consortium. **n**, Metadata of cardiac snRNA-seq, including XCI status assessment. **o**, ARs of X-linked genes across snRNA-seq pseudobulks. **p**, ATAC peak count analysis of adult and aged kidney and liver. **q**, ARs of X-linked ATAC peaks in adult and aged kidney. **r**, Human escape comparison. **s**, Reagents for cardiac retrograde perfusion. **t**, List of antibodies and dyes

Supplementary Table 2 Aging escape atlas.

## Data Availability

All datasets have been submitted to the Gene Expression Omnibus (GEO) database and can be accessed under the accession code GSE274695. All other data are available from the corresponding author upon request. In addition, we provide access to the escape landscape resource via the Integrative Genomics Viewer at https://github.com/AndergassenLab/AgingX.

## Code availability

The relevant code to reproduce the analyses and figures of this study is available via GitHub at https://github.com/AndergassenLab/AgingX.
