# Supplementary material for: Aging promotes reactivation of the Barr body at distal chromosome regions
Source: Nat Aging. 2025 May 1;5(6):984–96. doi: 10.1038/s43587-025-00856-8 (PMC12176624; doi:10.1038/s43587-025-00856-8)
Supplement: Supplementary file 1 — Reporting Summary [file 43587_2025_856_MOESM1_ESM.pdf]

nature portfolio

Corresponding author(s): Daniel Andergassen

Last updated by author(s): Jan 29, 2025

Reporting Summary

Nature Portfolio wishes to improve the reproducibility of the work that we publish. This form provides structure for consistency and transparency in reporting. For further information on Nature Portfolio policies, see our Editorial Policies and the Editorial Policy Checklist.

Statistics

For all statistical analyses, confirm that the following items are present in the figure legend, table legend, main text, or Methods section.

n/a	Confirmed	
☐	☒	The exact sample size (*n*) for each experimental group/condition, given as a discrete number and unit of measurement
☐	☒	A statement on whether measurements were taken from distinct samples or whether the same sample was measured repeatedly
☐	☒	The statistical test(s) used AND whether they are one- or two-sided<br>*Only common tests should be described solely by name; describe more complex techniques in the Methods section.*
☒	☐	A description of all covariates tested
☐	☒	A description of any assumptions or corrections, such as tests of normality and adjustment for multiple comparisons
☐	☒	A full description of the statistical parameters including central tendency (e.g. means) or other basic estimates (e.g. regression coefficient) AND variation (e.g. standard deviation) or associated estimates of uncertainty (e.g. confidence intervals)
☐	☒	For null hypothesis testing, the test statistic (e.g. *F*, *t*, *r*) with confidence intervals, effect sizes, degrees of freedom and *P* value noted<br>*Give P values as exact values whenever suitable.*
☒	☐	For Bayesian analysis, information on the choice of priors and Markov chain Monte Carlo settings
☒	☐	For hierarchical and complex designs, identification of the appropriate level for tests and full reporting of outcomes
☒	☐	Estimates of effect sizes (e.g. Cohen's *d*, Pearson's *r*), indicating how they were calculated

Our web collection on statistics for biologists contains articles on many of the points above.

Software and code

Policy information about availability of computer code

Data collection	No software was used for data collection.
Data analysis	Bowtie (version 2.3.5.1)<br>HTSeq (version 0.11.3)<br>Bedtools (version 2.30.0)<br>GATK (version 4.2.2.0)<br>Macs2 (version 2.1.0)<br>Sinto (version 0.9.0)<br>STAR (version 2.6.0c)<br>FastQC (version 0.11.9)<br>Cellranger (version 7.1.0)<br>Cellbender (version 0.3.0)<br>SNPsplit (version 0.6.0)<br>Seurat (version 5.1.0)<br>sctransform (version 0.4.1)<br>DoubletFinder (version 2.0.3)<br>Allelome.PRO (version 2 = Allelome.PRO2)

For manuscripts utilizing custom algorithms or software that are central to the research but not yet described in published literature, software must be made available to editors and reviewers. We strongly encourage code deposition in a community repository (e.g. GitHub). See the Nature Portfolio guidelines for submitting code & software for further information.

Data

Policy information about availability of data

All manuscripts must include a data availability statement. This statement should provide the following information, where applicable:

- Accession codes, unique identifiers, or web links for publicly available datasets
- A description of any restrictions on data availability
- For clinical datasets or third party data, please ensure that the statement adheres to our policy

All generated data have been submitted to the Gene Expression Omnibus (GEO) database under the accession code GSE274695.

Used publicly available annotation / databases:
GRCm38/mm10 RefSeq gene annotation (downloaded in 2018): O'Leary et al., 2016
Sanger database v5: Keane et al., 2011
ENCODE blacklist genes v2 Amemiya et al., 2019
International Mouse Phenotyping Consortium (IMPC): Groza et ., 2023
Alliance of Genome Resouces v7.2.0: Alliance of Genome Resources Consortium, 2024

Research involving human participants, their data, or biological material

Policy information about studies with human participants or human data. See also policy information about sex, gender (identity/presentation), and sexual orientation and race, ethnicity and racism.

Reporting on sex and gender	N/A
Reporting on race, ethnicity, or other socially relevant groupings	N/A
Population characteristics	N/A
Recruitment	N/A
Ethics oversight	N/A

Note that full information on the approval of the study protocol must also be provided in the manuscript.

Field-specific reporting

Please select the one below that is the best fit for your research. If you are not sure, read the appropriate sections before making your selection.

☒ Life sciences ☐ Behavioural & social sciences ☐ Ecological, evolutionary & environmental sciences

For a reference copy of the document with all sections, see nature.com/documents/nr-reporting-summary-flat.pdf

Life sciences study design

All studies must disclose on these points even when the disclosure is negative.

Sample size	No sample size calculations were performed. Sample sizes were determined based on previous experiments (Andergassen et al., 2017, Andergassen et al.,2021).
Data exclusions	No data were excluded.
Replication	For the comprehensive bulk RNA sequencing study, we used three biological replicates for each group (3 females, 3 males per postnatal timepoint; 3 females for embryonic timepoint). This number of replicates is generally used for RNA-seq in tissues from inbred mice, as low biological and technical variability between samples is expected. To test the variability in our samples, we performed unsupervised clustering (Spearman correlation) and were able to confirm the identity of the tissues by showing that replicates of the same tissue clustered together. This result implies a low biological and technical variability among the samples, which supports our choice of at least 3 biological replicates. For the single-cell heart analysis, which we performed to identify cell-type specificity of whole heart age-specific escapees, we performed an allele-specific analysis using the Allelome.PRO approach, which has the advantage of higher statistical power, because the expression signals of the two alleles serve as internal controls for each other, allowing robust confirmation of escapees from a single Adult and Aged replicate. For ATAC-seq, 2 biological replicates were used per organ and timepoint. For RNA-seq and ATAC_seq, all attempts at replication were successful.
Randomization	No randomization was performed. We used genetically identical inbred mice, eliminating the need for randomization to control genetic variability. Since age was our primary variable, mice were naturally assigned to groups based on chronological age, ensuring observed differences were due to aging rather than experimental bias.

Blinding	For in vivo sequencing data like in this study, blinding was not necessary, as the experimenter has no influence on the results of these tests, as the results are recorded directly by the machine.

Reporting for specific materials, systems and methods

We require information from authors about some types of materials, experimental systems and methods used in many studies. Here, indicate whether each material, system or method listed is relevant to your study. If you are not sure if a list item applies to your research, read the appropriate section before selecting a response.

Materials & experimental systems

n/a	Involved in the study	
☐	☒	Antibodies
☒	☐	Eukaryotic cell lines
☒	☐	Palaeontology and archaeology
☐	☒	Animals and other organisms
☒	☐	Clinical data
☒	☐	Dual use research of concern
☒	☐	Plants

Methods

n/a	Involved in the study	
☒	☐	ChIP-seq
☐	☒	Flow cytometry
☒	☐	MRI-based neuroimaging

Antibodies

Antibodies used	1. CD45 Monoclonal Antibody, PE-Cyanine5, eBioscience™, dilution 1:100 (Supplier: ThermoFisher, Catalog number: # 15-0451-81, Clone name: 30-F11, Lot number: 2228643)<br>2. PE Rat Anti-CD11b, dilution 1:100 (Supplier: BD Biosiences, Catalog number: # 553311, Clone name: M1/70, Lot number: 0301402)<br>3. PE/Dazzle™ 594 anti-mouse CD64 (FcγRI) Antibody, dilution 1:100 (Supplier: BioLegend, Catalog number: # 139320, Clone name: X54-5/7.1, Lot number: B304964)<br>4. F4/80 Monoclonal Antibody, PE-Cyanine7, eBioscience™, dilution 1:100 (Supplier: ThermoFisher, Catalog number: # 25-4801-82, Clone name: BM8, Lot number: 2198632)<br>5. CD140a (PDGFRA) Monoclonal Antibody, PE-Cyanine7, eBioscience™, dilution 1:50 (Supplier: ThermoFisher, Catalog number: # 25-1401-82, Clone name: APA5, Lot number: 2504580)<br>6. CD105 (Endoglin) Monoclonal Antibody, PE, eBioscience™, dilution 1:50 (Supplier: ThermoFisher, Catalog number: # 12-1051-82, Clone name: MJ7/18, Lot number: 2196698)<br>7. BD Pharmingen™ Purified Rat Anti-Mouse CD16/CD32 (Fc-block), dilution 1:50 (Supplier: BD Biosiences, Catalog number: # 553142, Clone name: 2.4G2, Lot number: 0296888)
Validation	All antibodies were validated by the supplier.<br>Suppliers' statements:<br>1. CD45: The 30-F11 antibody has been tested by flow cytometric analysis of mouse bone marrow cells and splenocytes. This can be used at less than or equal to 0.06 µg per test.<br>2. CD11b: Expression of CD11b on bone-marrow myeloid cells. BALB/c bone-marrow leukocytes were either unstained or stained with PE Rat Anti-CD11b (Cat. No. 553311/557397/561689). Flow cytometry was performed on a BD FACScan™ flow cytometry system.<br>3. CD64: Each lot of this antibody is quality control tested by immunofluorescent staining with flow cytometric analysis. C57BL/6 mouse bone marrow cells were stained with CD11b (clone M1/70) APC and CD64 (clone X54-5/7.1) PE/Dazzle™ 594 (top) or mouse IgG1, κ PE/Dazzle™ 594 isotype control (bottom).<br>4. F4/80: This BM8 antibody has been tested by flow cytometric analysis of mouse resident peritoneal exudate cells. This can be used at less than or equal to 0.5 µg per test.<br>5. CD140a: This APA5 antibody has been tested by flow cytometric analysis of NIH/3T3 cells. This can be used at less than or equal to 0.125 µg per test.<br>6. CD105: This MJ7/18 antibody has been tested by flow cytometric analysis of mouse brain-derived endothelial (bEnd.3) cells and splenocytes. This can be used at less than or equal to 0.5 µg per test.<br>7. CD15/CD32 (Fc-block):

Animals and other research organisms

Policy information about studies involving animals; ARRIVE guidelines recommended for reporting animal research, and Sex and Gender in Research

Laboratory animals	BL6 Xist -/+ females were purchased from Riken BRC Japan (RBRC02655: B6;129-Xist<tm5Sado>). C57BL/6J (BL6), and CAST/Ei (CAST) mice were purchased from the Jackson Laboratory.<br>F1 hybrids (female: BL6ΔXistxCAST, BL6xCAST; male: BL6xCAST) were used throughout the entire study. Experimental animals were sacrificed at E14.5, 4 weeks, 9 weeks, and 1.5 years of age.
Wild animals	This study did not involve wild animals.
Reporting on sex	Since the investigation of genes escaping female X chromosome inactivation and their expression levels compared to males was the

Reporting on sex	major aim of this study, we performed sex-based analyses.
Field-collected samples	This study did not involve samples collected from the field.
Ethics oversight	All animal experiments were performed in accordance with relevant guidelines and regulations, including the EU guideline 2010/63, the German Animal Welfare Act, and ARRIVE guidelines. Approval was granted by the authorities (District Administration Department of the City of Munich, Veterinary Office Munich City, permit according to §11, paragraph 1 sentence 1 no. 1 of the Animal Welfare Act).

Note that full information on the approval of the study protocol must also be provided in the manuscript.

Plants

Seed stocks	N/A
Novel plant genotypes	N/A
Authentication	N/A

Flow Cytometry

Plots

Confirm that:

☒ The axis labels state the marker and fluorochrome used (e.g. CD4-FITC).

☒ The axis scales are clearly visible. Include numbers along axes only for bottom left plot of group (a 'group' is an analysis of identical markers).

☒ All plots are contour plots with outliers or pseudocolor plots.

☒ A numerical value for number of cells or percentage (with statistics) is provided.

Methodology

Sample preparation	Sorting of major cardiac lineages (MCL)<br>To identify escape genes on cell-type resolution, hearts of 3 replicates per sex (BL6ΔXistxCAST females and BL6xCAST males) were isolated. In order to obtain a single cell suspension of the heart, an enzymatic retrograde perfusion was performed. To do so, the heart was harvested and cannulated at the aorta. Coronary arteries were flushed briefly with 1 ml Perfusion Buffer (reagents for buffer compositions see Supplementary Table 1, sheet p). Afterwards, the cannula with the heart was attached to the perfusion pump system, and the heart was perfused with Perfusion Buffer for 1 min (4 ml/min). Next, the heart was rinsed with a Digestion Buffer for 10 min (recirculating, 4 ml/min) to enzymatically dissociate the ventricular cells. Then, the heart was detached, atria were discarded, and the ventricles were roughly homogenized in 2.5 ml Digestion Buffer by cutting with scissors. A 1 ml syringe was used to shear the tissue pieces up and down for 30 times. After adding 2.5 ml Stop Buffer, the syringe sheared the liquid again for 30 times. Ultimately, the suspension was filtered through a 100 µm strainer and put on ice.<br>To separate cardiomyocytes from non-cardiomyocytes, the suspension was centrifuged at 100 x g at 4 °C for 1 min and supernatant was transferred. This was repeated once and the sediment, containing cardiomyocytes, was resuspended in 500 µl TRIzol, homogenized using a pellet pestle. After adding another 500 µl TRIzol the cardiomyocytes were frozen at -80 °C for further use. The supernatant, containing non-cardiomyocytes, was spinned down at 400 x g 4 °C for 7 min. After discarding the supernatant, the pellet was resuspended in RBC-Lysis-Buffer at room temperature for 0.5 min. Next, 2 ml PBS was added, and suspension was centrifuged at the same settings, followed by the filtering through a 70 µm strainer. Cells were blocked with Fc-block at 4 °C for 15 min, before using CD45-conjugated microbeads (Miltenyi) to bind CD45-positive cells. Afterwards, the cell suspension was separated into a CD45-positive and CD45-negative fraction using the autoMACS Pro Separator by Miltenyi. Then, both fractions were stained with Zombie Green to detect dead cells, followed by the macrophage staining for the CD45-positive fraction (CD45-positive, CD11b, CD64, F4/80) and the endothelial staining (CD45-negative, CD105) and the fibroblast staining (CD45-negative, CD140a) for the CD45-negative fraction. After washing twice, 2,000 cells of each cell type were sorted, immediately frozen on dry ice, and stored at -80 °C for further use.<br><br>Sorting of nuclei for 10X single-nucleus RNA-seq<br>To isolate nuclei from frozen tissue, the Nuclei Extraction Buffer (Miltenyi) was used according to manufacturer's instructions. For snRNA-sequencing, nuclei of 1 replicate per time point (BL6xCAST females) were incubated with DRAQ7 for 5 min before sorting, staining the DNA of nuclei. Sorting the DRAQ7-positive population ensured a debris-free nuclei suspension, which was assessed on the Countess™ II FL Automated Cell Counter equipped with the DAPI EVOS light cube.
Instrument	Sony Cell Sorter (make: SONY, Model number: LE-SH800SZGCPL)

Software	SH800 Software
Cell population abundance	In test experiments, sorted solution was observed und the microscope and counted by the Countess 3 Automated Cell Counter (Invitrogen) as well as manually. All cells were singlets, no dead cells, no debris was observed. In real experiments, 2,000 cells of each cell type were sorted, immediately frozen on dry ice, and stored at -80 °C for further use.
Gating strategy	Sorting of major cardiac lineages (MCL)<br>CD45-positive samples: The first gate excluded debris by considering cells of reasonable size on the FSC-A:BSC-A plot. Subsequently, a gate on the FSC-A:FSC-H plot was employed to exclude doublets. The Zombie-Green histogram then gated our sample into dead cells and living cells. The Zombie-Green-negative gate, representing living cells, was visualized on an CD45:CD11b plot. Population positive for both singals was gated. Ultimately, in a CD64:F4/80 plot the population positive for both singals was sorted as macrophages.<br>CD45-negative samples: The first gate excluded debris by considering cells of reasonable size on the FSC-A:BSC-A plot. Subsequently, a gate on the FSC-A:FSC-H plot was employed to exclude doublets. In the CD45:Zombie-Green plot, the population negative for both signals was gated and visualized on an CD105:CD140a plot. Population positive for CD105 was sorted as endothelial cells and population positive for CD140a wassorted as cardiac fibroblasts.<br><br>Sorting of nuclei for 10X single-nucleus RNA-seq<br>The first gate excluded debris by considering cells of reasonable size on the FSC-A:BSC-A plot. Subsequently, a gate on the FSC-A:FSC-H plot was employed to exclude doublets. In a DRAQ-7 histogram, the positive population was sorted as nuclei for single-nucleus libraries.

☒ Tick this box to confirm that a figure exemplifying the gating strategy is provided in the Supplementary Information.
